# A comparative analysis of cardiac bridging Integrator 1 (cBIN1) levels in patients with heart failure and healthy controls: A prospective case-control study

**DOI:** 10.12669/pjms.42.9.16094

**Published:** 2026-09

**Authors:** Azhar Iqbal, Ruqaya Nangrejo, Muhammad Nauman Khan, Qamar Aziz

**Affiliations:** 1 Dr. Azhar Iqbal, MBBS, M.Phil (Physiology), Diploma in Hematology, Post Graduate Student, Department of Physiology, Baqai Medical University, Karachi, Pakistan; 2 Dr. Ruqaya Nangrejo, MSc, PhD (Physiology). Professor of Physiology and PhD Supervisor, Baqai Medical University, Karachi, Pakistan; 3 Dr. Muhammad Nauman Khan, MBBS, FCPS (Cardiology). Associate Professor of Cardiology, National Institute of Cardiovascular Diseases / Jinnah Postgraduate Medical Centre, Karachi, Pakistan; 4 Dr. Qamar Aziz, MBBS, PhD (Physiology). Head and Chair, Professor of Physiology, Dept. of Physiology, Baqai Medical University, Karachi, Pakistan

**Keywords:** Biomarker, Cardiac Bridging Integrator 1, Ejection Fraction, Heart Failure

## Abstract

**Objectives:**

To compare cardiac bridging integrator 1 (cBIN1) levels between healthy individuals and patients with heart failure (HF), and to assess changes in cBIN1 levels following therapeutic intervention to evaluate its potential as a diagnostic and prognostic biomarker.

**Methodology:**

A case-control study with three months follow-up was conducted in the Department of Physiology, Baqai University Hospital, Karachi, Pakistan, from June 2024 to November 2024. A total of 200 adults aged 40-70 years were recruited, comprising 50 healthy controls with left ventricular ejection fraction (LVEF ≥60%) and 150 patients with heart failure (HF): Heart failure with reduced, moderatly reduced and preserved ejection fraction, HFrEF (LVEF ≤40%), HFmrEF (LVEF 40-49%) and HFpEF (LVEF ≥50%). Enzyme linked immuno-essay (ELISA) was used to measure the plasma cBIN1 level at baseline. For the statistical analysis, ANOVA and t-tests, as well as post-hoc testing with significance at p<0.05, were used.

**Results:**

Mean plasma cBIN1 was 9.221±0.440 ng/mL in healthy controls, 1.193±0.059 in HFpEF, 1.009±0.063 in HFmrEF and 0.799±0.050 in HFrEF (p<0.001), with the lowest levels in HFrEF on post-hoc testing (p<0.001 for all comparisons against controls). Levels were significantly lower in participants with higher BMI (3.19±3.65 vs 0.99±0.20; t=2.084, p=0.038) and did not differ significantly by gender (p>0.05). No significant change was observed after three months of follow-up (p=0.963).

**Conclusion:**

Plasma cBIN1 levels were significantly lower in patients with heart failure than in healthy controls, with the lowest levels observed in HFrEF. These findings suggest that cBIN1 may have value in stratifying HF severity and warrant evaluation in larger, multicenter cohorts.

## INTRODUCTION

Heart failure (HF) is among the top causes of morbidity and premature death in the world^1^, whose prevalence in the low-/middle-income countries is growing at an alarming pace, with late prevention and detection.^2^

Some of the influencing factors that have been increasing in South Asia, causing the increase in prevalence of HF, are urbanisation, ageing population, diabetes, high blood pressure, obesity, tobacco use, and air pollution.^3^,^4^ Not well-reported HF is also common among the Pakistani population, and high rates of acute decompensation, rehospitalization, and short-term mortality are not well-reported; therefore, improved risk stratification instruments are required in the normal care.^5^At the cellular level, HF development is linked directly to the impairment of excitation-contraction coupling and abnormal calcium-cycling proteins, channels, and nanoscale structures^6^, and depends on the state of the transverse-tubule (T-tubule) network and the nanoscale structure of ion channels and calcium-cycling proteins.^7^ One of the important regulators of T-tubule microfold stabilization, calcium influx, and sarcoplasmic reticulum calcium release coordination^8^ is cardiac bridging integrator 1 (cBIN1), an isoform of BIN1 localized to the cardiomyocytes, which is slowly expressed in the escalating HF severity.^9^

The new clinical research findings showed that plasma cBIN1 also differentiates HF phenotypes and provides prognostic value as a patient-in-hospital factor; thus, plasma cBIN1 can be used to determine severity and predict prognosis.^10^Access to HF detection pathways may be enhanced via biological detectors, such as cBIN1,^11^ and preventive interventions in both diabetes and obesity, which could contribute to increasing the rates of early diagnosis, enhancing treatment, and reducing mortality rates related to the case of HF.^12^

So far, cBIN1 has only been studied as a biomarker in Chinese and American cohorts, so it’s unclear whether plasma cBIN1 levels behave the same way in South Asians, a population with a genetically distinct and disproportionately high cardiovascular risk profile.^`13^ Heart failure research in Pakistan still relies mainly on conventional echocardiographic and clinical parameters, with no local evaluation of a non-invasive myocardial remodeling marker like cBIN1.^2^ This gap is compounded by South Asia’s limited biobanking infrastructure for validating emerging biomarkers.^14^ Meanwhile, heart failure is a growing problem in Pakistan given the high burden of hypertension, diabetes, and obesity, making an accessible, reliable diagnostic tool genuinely needed.^14^ A Karachi-based study measuring plasma cBIN1 levels would help establish whether this biomarker holds diagnostic value locally and add much-needed South Asian representation to the HF biomarker literature.

The present study aimed to find out the level of the predictive biomarker of the severity of HF, cBIN1, in the Pakistani population, and whether the level of cBIN1 is related to the severity of the clinical picture. The short-term outcomes will also be determined to enhance risk stratification and utilization of the risks in preventive clinical decision-making.

Primary objective of the study was to determine and compare plasma cBIN1 levels in patients with heart failure and in healthy controls, and to examine their association with heart failure phenotype. Secondary objectives was to assess the change in plasma cBIN1 after three months of guideline-directed medical therapy, and to examine the association of cBIN1 with age, gender, BMI and left ventricular ejection fraction.

## METHODOLOGY

The research was conducted at Baqai University Hospital, Karachi, Pakistan, June 2024 to November 2024. The proposed case-control study was a prospective one comparing the levels of cBIN1 in healthy adults and patients having heart failure (HF).

### Ethical approval:

The research received the approval of the Institutional Review and Ethics Board, Baqai Medical University (Ref: BMU-IREB/05-2023 dated: November 20, 2023). It was performed in conformity with the facts of the Declaration of Helsinki and that all the members of the study signed informed consent in the designed proforma.

### Participants

The participants were classified based on the criteria for diagnosis developed by the European Society of Cardiology (ESC) and the American Heart Association (AHA).^15^ Controls were not matched to patients; baseline differences were adjusted for in the multivariable regression analysis.

### Inclusion criteria:

The participants were recruited into the study as adults of either sex, between the ages of 40-70 years, with a presentation of congestive heart failure. Comparatively, healthy adults of the same age group were recruited.

- Adults aged 40-70 years
- Female and male participants.
- Healthy adults as control

### Exclusion criteria:

The exclusion criteria for heart failure participants were:

- Pregnant women
- Severe Chronic obstructive lung disease (COPD)
- Hepatitis or chronic renal disease.
- Any malignancy
- Myocarditis, myopathy, or valvular cardiac disease.
- Heart bypass surgery (coronary bypass, replacement of the heart valves, or replacement) within six months.

### Study protocols:

All participants underwent a standardized baseline assessment comprising clinical history, physical examination, 12-lead electrocardiography, transthoracic echocardiography and routine laboratory investigations (complete blood count, renal and hepatic profile, fasting glucose and lipid profile). The diagnosis and phenotypic classification of HF were made according to the 2021/2023 ESC criteria. Plasma cBIN1 was measured at two time points: at enrolment, before initiation or optimization of guideline-directed medical therapy (baseline), and after three months of follow-up (post-intervention).

### Study groups:

The groups of the study were formed based on the principles of the European Society of Cardiology (ESC).^13^

### Group-A (Healthy Controls):

Fifty healthy individuals who had a left ventricular ejection fraction of ≥ 60 per cent. were included in this group.

***Group-B (Severely Reduced Ejection Fraction - HFrEF):*** Fifty patients with LVEF ≤40.

***Group-C (Moderately Reduced Ejection Fraction - HFmEF):*** Fifty participants whose LVEF was between 40-49.

***Group-D (Preserved Ejection Fraction - HFpEF):*** LVEF ≥50.

### Group-E (Post Intervention Group):

Seventy five participants (Groups B, C and D who had received three months of intervention treatment.

Intervention treatment: Initially, all 150 participants with heart failure were instructed to take their prescribed medications regularly and attend monthly follow-up visits throughout the study period. At the end of the study, participants were classified according to their level of adherence. The 75 participants who consistently followed the prescribed treatment regimen and attended all scheduled monthly follow-up visits were categorized as the compliant (intervention) group. The remaining 75 participants who were unable to adhere to the prescribed treatment and/or failed to attend the scheduled monthly follow-up visits were categorized as the non-compliant group (Group E post-intervention group).

### Compliant Group:

(n =75) The group comprises participants with heart failure who had previously used the therapeutic guidelines for three months and regularly visited.

### Non-Compliant Group:

(n = 75) It comprises heart failure participants who were unable to follow the therapeutic regimen and follow-up protocols.

### Sampling and Sample size:

Based on previous cBIN1 research by Nikolova et al.,^16^ approximately 50 participants were included in each study group. A total of 200 participants were enrolled, comprising 150 patients with heart failure (50 each with HFrEF, HFmrEF, and HFpEF) and 50 apparently healthy controls, giving a 3:1 case-to-control ratio. At a two-sided α of 0.05 and 80% power, this sample size was sufficient to detect a standardized mean difference (Cohen’s d) of approximately 0.46 between heart-failure patients and controls.

### Measures and surveys:

The commercial ELISA kits (Cloud-Clone) were also used to determine the phasic cBIN1 outcome, but in this case, the lab personnel were unaware of the clinical findings, hence limiting the bias. The measurement of LVEF was performed with the assistance of Echocardiography. Plasma cBIN1 was quantified using a commercial sandwich ELISA kit (Cat. No. SEJ555Hu, Cloud-Clone Corp., Wuhan, China) strictly according to the manufacturer’s instructions. The assay detection range was 0.156–10-ng/mL with a minimum detectable dose of <0.055-ng/mL; intra-assay and inter-assay coefficients of variation were <10% and <12%, respectively. All samples were assayed in duplicate by laboratory personnel blinded to participants’ clinical and echocardiographic data.

**Table-I:** Baseline demographic, clinical, and laboratory characteristics by study group (N = 200).

Characteristic	HFrEF (n = 50)	HFmrEF (n = 50)	HFpEF (n = 50)	Healthy controls (n = 50)	F	p-value
Age, years	51.62	50.22	52.66	43.34	21.816	<0.001
Body mass index, kg/m²	22.32	22.81	22.64	22.74	1.117	0.343
Systolic blood pressure, mmHg	128.06	132.10	142.90	118.38	22.413	<0.001
Diastolic blood pressure, mmHg	81.66	84.70	90.16	79.60	16.230	<0.001
Left ventricular ejection fraction, %	32.60	42.20	53.10	64.80	1137.542	<0.001
Haemoglobin, g/dL	12.742	12.312	12.144	13.142	3.721	0.012
Urea, mg/dL	27.78	27.04	25.32	15.20	67.940	<0.001
Creatinine, mg/dL	1.162	1.090	1.100	0.980	15.279	<0.001
HbA1c, %	6.318	6.368	6.406	6.272	1.235	0.298
Total cholesterol, mg/dL	199.22	200.60	201.60	199.76	0.681	0.565
Triglycerides, mg/dL	127.80	133.78	130.88	123.76	7.730	<0.001
LDL cholesterol, mg/dL	103.86	115.26	107.86	100.78	10.993	<0.001
HDL cholesterol, mg/dL	43.38	41.86	41.90	46.86	7.892	<0.001
ALT (SGPT), U/L	24.94	21.82	23.34	22.08	1.219	0.304
AST (SGOT), U/L	15.20	15.06	16.36	14.46	2.006	0.114
GGT, U/L	26.14	26.58	25.96	25.42	0.889	0.448
Alkaline phosphatase, U/L	78.92	75.68	75.98	80.82	3.899	0.010
Male sex, n (%)	30 (60.0)	25 (50.0)	27 (54.0)	27 (54.0)	0.338	0.798

Values are group means unless otherwise stated; between-group comparison by one-way ANOVA.

ALT: alanine aminotransferase; AST: aspartate aminotransferase; GGT: gamma-glutamyl transferase; HbA1c: glycated haemoglobin; HDL: high-density lipoprotein; HFmrEF: heart failure with mildly reduced ejection fraction; HFpEF: heart failure with preserved ejection fraction; HFrEF: heart failure with reduced ejection fraction; LDL: low-density lipoprotein.

**Table-II:** Plasma cBIN1 concentration by study group at baseline and at three months, with one-way ANOVA.

Group	n	Baseline cBIN1, mean (ng/mL)	Baseline 95% CI	Three-month cBIN1, mean (ng/mL)	Three-month 95% CI	p-value
HfrEF	50	0.799	0.736 to 0.862	0.800	0.737 to 0.863	-
HfmrEF	50	1.009	0.946 to 1.072	1.004	0.941 to 1.067	-
HfpEF	50	1.193	1.130 to 1.256	1.198	1.135 to 1.261	-
Healthy controls	50	9.221	9.158 to 9.284	9.221	9.158 to 9.284	-
All participants	200	3.056	2.557 to 3.554	3.056	2.557 to 3.554	-
Between groups	200	F = 16591.07	η² = 0.996	F = 16645.83	η² = 0.996	<0.001

Group means are the Tukey homogeneous-subset means from the SPSS output. Confidence intervals are model-based, derived from the ANOVA within-groups mean square (0.051, df = 196;

pooled SD 0.226). Baseline: between-groups SS 2538.196, df 3; within-groups SS 9.995, df 196.

Three months: between-groups SS 2538.085, df 3; within-groups SS 9.962, df 196. Healthy controls were sampled once only; the three-month column reproduces their single measurement and they are excluded from all paired analyses. cBIN1: cardiac bridging integrator 1; CI: confidence interval; η²: eta-squared.

### Data collection procedures:

Informed consent was obtained from the participants. Plasma was extracted from blood and preserved at -80°C until thawed for detection of cBIN1.

### Statistical analysis:

Data analysis was done using SPSS 25. The continuous variables were summarized as a mean and a deviation between standard deviation (SD), and the number of counts as well as percentages were represented and reported for the categorical variables. The statistical tests were an independent-samples t-test, chi-square tests, paired t-tests, and ANOVA. Significance was set at p < 0.05. No sensitivity analyses were performed.

## RESULTS

Of the 200 participants enrolled (150 patients with HF and 50 healthy controls), all provided baseline samples. Repeat samples after three months were available from all 150 HF patients. Post-follow-up analyses are therefore restricted to this subgroup and are reported separately from the baseline analyses of the full cohort. Participant flow: 200 adults were enrolled (150 HF patients, 50 healthy controls). All 200 provided baseline samples. All 150 HF patients completed the three-month follow-up and provided repeat samples. No participants were lost to follow-up.

**Table-III:** Post-hoc pairwise comparisons of plasma cBIN1 between study groups (Tukey HSD).

Comparison	Mean difference	95% CI	Standard error	Cohen’s d	p-value
HFrEF vs HFmrEF	−0.210	−0.327 to −0.093	0.045	0.93	<0.001
HFrEF vs HFpEF	−0.393	−0.510 to −0.276	0.045	1.74	<0.001
HFrEF vs healthy controls	−8.422	−8.539 to −8.305	0.045	37.29	<0.001
HFmrEF vs HFpEF	−0.183	−0.300 to −0.066	0.045	0.81	<0.001
HFmrEF vs healthy controls	−8.212	−8.329 to −8.095	0.045	36.36	<0.001
HFpEF vs healthy controls	−8.029	−8.146 to −7.912	0.045	35.55	<0.001

Baseline values. Each pair appears once; the reciprocal duplicates present in the original submission have been removed, and the previously missing HFpEF versus healthy controls comparison has been added. A negative mean difference indicates a lower cBIN1 concentration in the first-named group. Cohen’s d is calculated from the pooled within-group SD (0.226). Tukey HSD controls the family-wise error rate; the Bonferroni-adjusted comparisons in the SPSS output gave the same conclusions. cBIN1: cardiac bridging integrator 1; CI: confidence interval.

The overall mean pre-intervention cBIN1 level was 3.06 ± 3.58 (range: 0.71–9.98), while the post-intervention mean was 3.06 ± 3.58 (range: 0.70–9.98), showing no significant overall change after three months of therapy.

One-way ANOVA demonstrated highly significant differences in cBIN1 levels among study groups at both baseline and post-intervention assessments (pre-intervention: F=16591.07, P<0.001; post-intervention: F=16643.90, P<0.001). Within-group variability was minimal (mean square=0.051).

Post-hoc Tukey analysis revealed that the HFrEF group had significantly lower baseline cBIN1 levels compared to HFmEF (mean difference −0.21), HFpEF (−0.39), and healthy controls (−8.42) (P<0.001). Similarly, HFmEF and HFpEF groups had significantly lower levels compared to controls (P<0.001). Comparable differences were observed at post-intervention evaluation, with HFrEF demonstrating the lowest cBIN1 levels (P<0.001).

Participants were categorized into four age groups: 31–40 years (n=23), 41–50 years (n=94), 51–60 years (n=73), and 61–70 years (n=10). Baseline mean cBIN1 levels ranged from 3.44 to 4.11, with the highest mean observed in the 51–60 years group (4.11 ± 3.68).

ANOVA showed statistically significant differences among age groups at both baseline (F=521.17; P<0.001) and post-intervention (P<0.001). However, paired t-test analysis within each age group demonstrated no significant pre- and post-intervention change (all P>0.05).

Participants were divided into two BMI categories (Category 1: n=188; Category 2: n=12). Baseline cBIN1 levels were significantly higher in Category 1 (3.19 ± 3.65) compared to Category 2 (0.99 ± 0.20) (t=2.084; P=0.038). Similar findings were observed post-intervention (3.19 ± 3.65 vs. 1.01 ± 0.17; t=2.057; P=0.041).

No statistically significant differences in cBIN1 levels were observed between males and females at baseline or post-intervention (P>0.05). Additionally, no significant intra-gender changes were noted following therapy.

## DISCUSSION

This study evaluated circulating bridging integrator 1 (cBIN1) levels in healthy individuals and patients with heart failure (HF) and examined the impact of a three-month observational therapy consisting of pharmacological management and lifestyle modification on cBIN1 levels and left ventricular ejection fraction (LVEF). The principal findings demonstrate that cBIN1 levels were significantly reduced in all HF phenotypes compared with healthy controls, with the lowest levels observed in patients with heart failure with reduced ejection fraction (HFrEF). Although LVEF improved following therapy, cBIN1 levels did not show significant change over the short-term follow-up period. The marked reduction in circulating cBIN1 levels across HF phenotypes,^10^ is consistent with the established role of BIN1 in maintaining transverse-tubule (T-tubule) integrity and excitation–contraction coupling in cardiomyocytes.^17^ Structural remodeling of T-tubules disrupts calcium handling, contributing to impaired systolic contraction and diastolic relaxation. Prior experimental and clinical studies have shown that loss of BIN1 expression leads to defective calcium channel trafficking and abnormal excitation–contraction coupling, thereby promoting progressive myocardial dysfunction.^9^,^18^,^19^

**Table-IV:** Plasma cBIN1 at baseline and three months in heart failure patients by adherence status, and paired comparison.

Group	n	Baseline cBIN1 (mean ± SD)	Three-month cBIN1 (mean ± SD)	Mean change	95% CI of difference	p-value
Non-adherent	75	0.851 ± 0.087	0.867 ± 0.109	+0.016	-	-
Adherent	75	1.150 ± 0.079	1.135 ± 0.107	−0.015	-	-
Between-group difference, baseline	150	−0.299	-	-	−0.325 to −0.272	<0.001
Between-group difference, three months	150	-	−0.268	-	−0.302 to −0.233	<0.001
Paired baseline vs three months (all participants)	200	3.056 ± 3.578	3.056 ± 3.578	−0.0002	−0.0100 to 0.0095	0.963

Adherence was defined as attendance at all scheduled monthly reviews together with self-reported intake of ≥80% of prescribed doses. Between-group differences by independent-samples t-test (baseline t = −22.002, df = 148; three months t = −15.207, df = 148); Levene’s test was non-significant at both time points (p = 0.256 and p = 0.914). The paired comparison was by paired t-test (t = −0.046, df = 199). cBIN1: cardiac bridging integrator 1; CI: confidence interval; SD: standard deviation. ANOVA comparing levels of Pre and Post CBIN1 levels in HFrEF, HFmrEF, HFpEF, and Normal Control Group P<0.05*, P<0.001 **

Our findings suggest that reduced circulating cBIN1 reflects underlying structural myocardial remodeling rather than transient hemodynamic variation.^17^ The graded decline in cBIN1 levels-lowest in HFrEF, followed by HFmEF and HFpEF further supports its potential role as a marker of myocardial disease burden and structural integrity.^16^ This pattern aligns with the concept that progressive impairment of systolic function is associated with greater ultrastructural disruption within cardiomyocytes. Despite measurable improvement in LVEF after three months of therapy, cBIN1 levels remained unchanged. This dissociation suggests that cBIN1 is a relatively stable marker of chronic myocardial ultrastructural damage that may not respond to short-term, non-invasive therapeutic strategies. Unlike natriuretic peptides, which reflect acute hemodynamic stress and are sensitive to therapeutic optimization, cBIN1 appears to represent deeper structural remodeling processes.

Previous evidence indicates that recovery of BIN1 expression may require prolonged reverse remodeling or more advanced interventions such as cardiac resynchronization therapy or mechanical unloading.^20^ Therefore, the absence of significant short-term change in cBIN1 levels in our cohort does not negate its clinical relevance but rather reinforces its potential value as a marker of chronic structural myocardial health. An improvement in LVEF was observed particularly among patients with HFrEF and HFmEF. However, this functional improvement was not accompanied by changes in cBIN1 levels. This divergence underscores the multifactorial nature of myocardial recovery. Systolic performance can improve through modulation of preload, afterload, neurohormonal pathways, and myocardial energetics without complete reversal of ultrastructural remodeling. These findings support the emerging perspective that cBIN1 may provide complementary prognostic information beyond conventional echocardiographic indices.^13^,^16^ While LVEF reflects global ventricular function, cBIN1 may more specifically indicate cardiomyocyte structural integrity. Integration of structural biomarkers with imaging parameters may enhance risk stratification and long-term monitoring in HF populations.

Higher BMI was associated with lower cBIN1 levels in this study, highlighting the role of metabolic stress in myocardial structural integrity. Obesity is known to promote lipotoxicity, chronic inflammation, and oxidative stress, all of which may accelerate BIN1 depletion and T-tubule disruption.^10^ The persistence of reduced cBIN1 levels despite short-term lifestyle modification suggests that sustained metabolic control or longer intervention duration may be required to influence structural myocardial remodeling. Age-related differences were also observed, with relatively higher baseline cBIN1 levels in the 51–60-year age group. However, no significant pre- and post-intervention differences were detected across age categories. This finding may indicate that age influences baseline myocardial BIN1 expression,^13^ consistent with evidence of age-associated myocardial remodeling^20^ rather than short-term responsiveness to therapy.

The substantial difference in cBIN1 levels between HF patients and healthy controls underscores its potential as a discriminative biomarker of pathological myocardial remodeling. Unlike traditional biomarkers that may overlap across disease states, the magnitude of difference observed suggests strong diagnostic potential. Although no significant gender-based differences were identified, larger studies with adequate statistical power are warranted to explore potential sex-specific variations in cBIN1 expression and therapeutic response.

Collectively, these findings indicate that cBIN1 serves as a stable biomarker of myocardial structural integrity rather than a marker of acute therapeutic response. Its consistent reduction across HF phenotypes and relative insensitivity to short-term intervention suggest greater utility in disease classification, prognostication, and long-term follow-up.

### Limitations

The relatively modest sample size may limit generalizability of the findings. The short duration of follow-up precluded assessment of long-term effects on cBIN1 expression and cardiac remodeling. Additionally, adherence to lifestyle interventions was self-reported, which may introduce reporting bias. Larger, multi-center studies with longer follow-up are recommended. cBIN1 showed complete separation between groups, producing degenerate ROC statistics (AUC = 1.000 with standard error zero). Therefore, logistic regression and ROC analysis were not reported. This separation limits our ability to propose a clinical diagnostic cut-off; replication in an independent, multi-center cohort is required before any diagnostic threshold can be proposed.

## CONCLUSION

In this single-center observational study, plasma cBIN1 was significantly lower in patients with heart failure than in healthy controls, with the lowest levels observed in HFrEF and a graded pattern across phenotypes. Levels did not change measurably over three months of guideline-directed medical therapy. Because the separation in this dataset is complete, these findings do not establish diagnostic accuracy. They identify cBIN1 as a candidate marker of heart failure phenotype that warrants evaluation in larger, multicenter prospective cohorts.

### Authors’ Contribution:

**AI** conceived and designed the study, recruited participants, performed assays, analyzed and interpreted data, drafted the manuscript and is responsible for the integrity of the study.

**RN** supervised design and conduct and reviewed the analysis.

**MNK** supervised echocardiographic phenotyping and clinical classification.

**QA** supervised laboratory methodology.

All authors critically revised and approved the final version and agree to be accountable for all aspects of the work.
